# Is Income Inequality ‘Toxic for Mental Health’? An Ecological Study on Municipal Level Risk Factors for Depression

**DOI:** 10.1371/journal.pone.0092775

**Published:** 2014-03-27

**Authors:** Heikki Hiilamo

**Affiliations:** University of Helsinki, Department of Social Research, Helsinki, Finland; Wayne State University, United States of America

## Abstract

Most inequality research on the relationship between inequality and mental health has focused on cross-country variation. Findings from within-country data are mixed. We examined whether changes in municipal Gini index or in the share of people living in relative poverty were linked to changes in the use of antidepressants in several Finnish municipalities between 1995 and 2010. We found that more young adult females used antidepressants in municipalities where relative poverty had increased. Changes in municipal-level Gini index were not positively associated with changes in the use of antidepressants in the municipalities between 1995 and 2010. However, fewer elderly females used antidepressants in municipalities where the Gini index increased. In addition, more young adults used antidepressants in municipalities where the number of those not being educated or trained had also increased. An increase in the number of persons over 65 years of age living alone was positively associated with an increase in the use of antidepressants among elderly females.

## Introduction

The relationship between income inequality and poor health is one of public health science's best-known and most extensively researched topics. Yet there are no universal, unambiguous and generally accepted interpretations of cause-effect relationships between measures of income inequality and general measures of health [1],[2],[3],[4],[5],[6],[7]], or more specifically, between inequality and specific health outcomes such as mental illness [8],[9],[10],[11],[12],[13],[14].

At the individual level the case is clear: low socioeconomic status predicts mental health problems [15],[16],[17]. A study conducted with a low-income multi-ethnic sample of 98 families recruited from the greater Denver area in the US showed that poverty-related stress was directly related to symptoms of anxiety/depression [18]. The researchers labelled persistent poverty as “toxic for one's psychological health”.

At the macro level, two major theories have been presented to depict the general mechanisms of income inequality and health. According to the stress theory [2], [19], people constantly compare themselves with others. Low status in a community – whether it be English white-collar workers [2] or Masai Mara baboons [20] – often results in feelings of inferiority, increases stress and weakens health. The theory predicts that in rich countries the direct effects of income inequality represent generalizable or particular psychological processes that influence health outcomes [21]. Inequality may subject mental health to stress while putting out of reach many potential sources of social support [22]. It is also possible that wide social distances between people at the top of the income ladder cause stress and mental health problems.

The so-called materialist theory does not emphasize emotion and hormonal secretion, but rather the actual living conditions of the rich and poor [23], [24]. The theory assumes that people with low incomes do not necessarily fare worse because they feel oppressed and inferior: it could simply be that they have manifestly lower incomes and poorer services than those with higher incomes. Poor health is connected with a lack of resources, not with psychological processes. In an aggregate analysis, problems tend to accumulate in areas where poor people live simply because there are many such people.

One of the most important criteria for causality, in addition to statistical significance and strength, is the temporal relationship [25]. Neither of the mechanisms makes it clear what time span is involved in the materialization of the relationship between income inequality and potential health effects. Income inequality would have had to appear before the effects became apparent. It is perhaps a case of long-term “exposure” to a potential risk factor in the environment. However, lags in the associated effects of the various factors are most likely to be different as well, which makes things more complex. The question of exposure appears more difficult to handle in studies where long-term outcomes have been examined (e.g., suicide mortality as an indicator of mental health). In this study, we focus on a possibly more immediate health consequence of income inequality, namely depression. We calculate our final models for lags of one, two and tree year with regard to Gini index and relative poverty.

Mental health is a suitable candidate for a group-level health outcome affected by income inequality [10]. Income inequality relates to the degree to which material resources are disproportionately distributed. Another way to look at income inequality is to consider it a measure of the distance between the most privileged and the most deprived within a particular social group. In a study by Galea et al. [26], a neighbourhood's socioeconomic status (SES) was associated with the incidence of depression independent of individual SES and other individual covariates, which highlights the importance of taking account of the contextual risk factors for mental health.

Most research on the relationship between inequality and mental health has focused on cross-country variation [10], [12], [13] where cultural, social and institutional confounding factors may have distorted the results [12]. Previous findings from within-country data are mixed. An US study showed that the prevalence of depression was significantly associated with income inequality – the more unequal, the higher the prevalence of depression [14]. Another county-level study showed income inequality to be significantly associated with depression among older Americans [8]. Yet another comparative study of US states showed that the income inequality between them did not increase symptoms of depression [9].

The aim of this municipal-level study is to evaluate whether changes in municipal-level Gini coefficient or the share of people living in relative poverty are linked to changes in the use of antidepressants in Finnish municipalities between 1995 and 2000. With its 336 municipalities and marked regional heterogeneity, Finland provides an unusual opportunity to examine the putative association between income inequality and the use of antidepressants.

Broadly speaking, there are two basic ways to tackle income inequality as a measurement concept. The *Gini index or coefficient* (here values between 0 and 100) measures how income is distributed across a population within a defined geographical area, while the *relative poverty rate* gives the proportion of households whose income falls below a certain level (the poverty line) within a defined geographical area, excluding them from ordinary living patterns, customs and activities [27].

In this study the Gini index and relative poverty measure two different dimensions of inequality. The Gini index calculated for each municipality measures the distance between the richest and poorest households within the municipality. Insofar as people are expected to compare their standards of living with those of their peers in the municipality where they live, this measure can be taken as an operationalization of the stress theory. The effects of the municipal Gini coefficient are deemed to reflect negative emotions such as shame and distrust, which may be associated with disadvantage and perceptions of disadvantage that are claimed to be directly linked to depression.

The relative poverty rate calculated for each municipality using the national poverty threshold indicates the number of persons whose ability to participate in society is compromised according to a national standard. This measure can be used to test the validity of the materialist theory. While the Gini index measures the distances within the municipality, the relative poverty shows the share of people in the municipality who are left behind from all the others in the country.

**Figure 1:**
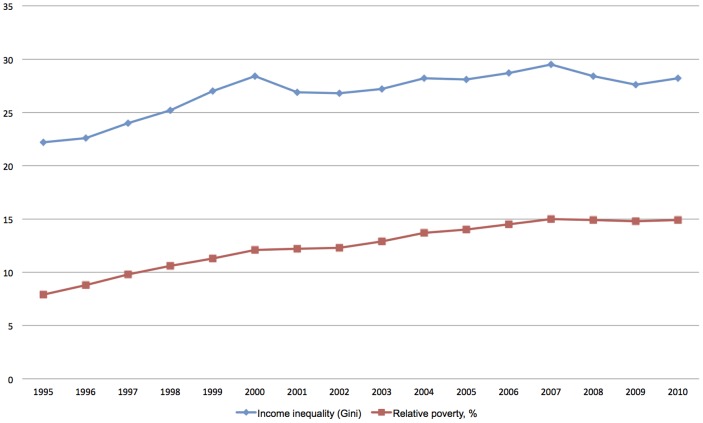
Gini index and relative poverty between 1995 and 2010 in Finland.

In the early 1990s Finland experienced a deep economic recession, later labelled the Great Depression [28]. Following this recession the gap between rich and poor widened in Finland more than in any other wealthy industrialized country at the time [29]. The national Gini coefficient rose from 22.2 in 1995 to 28.4 in 2000 and remained at that level until 2010 (Figure 1). In 1995 the relative poverty rate (60 per cent of median income) was 7.9 per cent while in 2000 it was 12.1 per cent. The rate continued to increase until 2010 but at a slower pace. The rapid increase in inequality from a very low level makes Finland an interesting case with regard to the health effects of income inequality [4], [30].

Social sciences deal with contexts that are highly complex, adaptive and not rigorously rule-bound. To gain a more accurate picture of the pathways through which material conditions affect health outcomes, it is also necessary to study their effect on various health outcomes according to age groups and gender [5].

## Data

Data were collected yearly from the SOTKANet statistics and indicator bank (www.sotkanet.fi) from 1995 to 2010. SOTKAnet contains comprehensive municipal-level statistical information on welfare and health in Finland. Whenever possible, the data were collected separately for both males and females in three age groups: young adults (18–24 years), working aged persons (24–64 years) and the elderly (65 years or older). Due to missing values and in order to reduce random variation in the dependent variable, municipalities with less than 1000 inhabitants were excluded from the analysis of working aged and elderly populations, while municipalities with less than 4000 inhabitants were excluded from the analysis of young adults (Table 1).

**Table 1:** Variables used in the analysis of Finnish municipalities between 1995 and 2010.

	N	Mean	StdD	Min	Max
Proportion of young (18–24 y) females using antidepressants, %	3514	4.18	2.29	0	14
Proportion of young (18–24 y) males using antidepressants, %	3514	2.47	1.28	0	7.7
Proportion of working-aged (25–64 y) females using antidepressants, %	5153	8.08	2.58	0.6	17.2
Proportion of working aged (25–64 y) males using antidepressants, %	5154	5.2	1.75	0.6	12.7
Proportion of elderly (65+y) females using antidepressants, %	5140	10.95	2.81	0.8	23.4
Proportion of elderly (65+y) males using antidepressants, %	5096	7.79	1.94	0	17.3
Income inequality (Gini)	5154	24.19	2.91	15.9	63.1
Relative poverty (60% poverty treshold). %	5154	14.21	4.74	3	29.9
Proportion of females with a higher education %	5154	18.48	7.16	5	55.8
Proportion of males with a higher education, %	5154	14.41	6.65	2.3	56.8
Proportion of young females (17–24 y) not in education or training, %	3514	9.45	2.99	0.5	25.4
Proportion of those young males (17–24 y) not in education or training, %	3514	13.59	3.82	4.3	30.3
Youth (16–24 y) unemployment (both male and female), %	3510	17.68	9.17	1.2	51.6
Divorces among those aged 25–64, per 1000 married persons	5154	12.0	5.0	0	35.0
Unemployment (female), %	5135	13.03	5.28	0.8	51.6
Unemployment (male), %	5135	12.91	5.98	1.1	40
Proportion of elderly (65+y) living alone, %	5152	47.13	5.33	26.5	69.1

The proportion of antidepressant users in a municipality was taken as a proxy for the prevalence of depression. In the SOTKAnet database, information on the use of antidepressants is derived from the prescription register of the Social Insurance Institution of Finland. The Social Insurance Institution is responsible for providing reimbursements for outpatient medical expenses. Well over 300 000 persons from a population of 5.4 million received reimbursements for prescription antidepressants in 2010. The medicines must be prescribed by a medical doctor. Nearly all outpatients on medication are included in the Social Insurance Institution's prescription register where antidepressants are represented by Anatomic Therapeutic Chemical (ATC) classification group N06A (http://www.whocc.no/atc_ddd_index/). The criteria for prescribing medication for depression may vary between physicians and geographical areas. Not all patients suffering from depression use antidepressants. At the municipal level, the use of antidepressants is affected not only by the number of persons suffering from depression but also by access to general practitioners, care practices and modes of operation. Antidepressants are prescribed for other conditions as well as depression, but depression remains the main indication for antidepressant use [31].

Data for the Gini coefficient in the SOTKAnet database were collected from the income distribution register. The coefficient gives the distribution of disposable income across households in the region under examination, in this case particular municipalities. The value ranges between 0 and 1. In the analysis, the figure was multiplied by 100. The greater the value, the more unequally is the income distributed.

Relative poverty was measured in the SOTKAnet database by the general at-risk-of-poverty rate of the municipality. The measure gives the proportion of persons in the municipality who live in households with incomes below the at-risk-of-poverty threshold. The at-risk-of-poverty threshold was set at 60 per cent of each year's median equivalent disposable income of all Finnish households (according to the adjusted OECD scale). This was recalculated each year based on the entire population's income distribution at the national level. Accordingly, the indicator describes the population having a low income by national standards as a proportion of the total population of the municipality. Both the Gini coefficient and the at-risk-of-poverty rate are relative concepts; the former takes account of the municipal income distribution, while the latter is affected by changes taking place in median income and incomes below the median in the distribution of national income.

Research on socio-economic health differences has shown that social status and health affect each other at the macro, meso and micro levels through a number of factors [4]. Given the known associations between depression and educational achievement [15], the models include a measure for education level. The indicator for educational level gives the gender-specific proportion of persons aged 15 and over with a higher education. Persons with a higher education refers to those who have completed, in a vocational institution, studies of more than 3 years leading to a vocational qualification, or those who have completed a polytechnic or university degree.

Additionally, we employ control variables for specific age groups. For young adults we have the proportion of those not being educated or trained in the 17–24 age group. The indicator ‘persons not being educated or trained or being employed’ (NEET) refers to those who were not students during the year in question or who did not earn a degree or other qualification after basic education. We also have the rate of unemployment among the population aged 16 to 24. The data draw on labour force surveys complying with guidelines issued by the Statistical Office of the European Communities (Eurostat) and the International Labour Organization (ILO). For the working aged population we controlled for gender-specific unemployment rates and divorces among those aged 25–64 per 1000 married persons of the same age. For the elderly we have households with one person aged 65 or over as a percentage of all households with persons of the same age.

## Methods

We used regression models where municipality is a fixed factor, and Gini coefficient, relative poverty, educational level and control variables were used as covariates. As additional control variables we used gender and age-specific unemployment, divorces, share of young people out of education and employment and share of elderly single households. The proportion of antidepressant users is a dependent variable. The models were analysed separately for the three age groups and both genders. A fixed effect method was chosen to emphasize the huge variation in the independent and dependent variables [32] and that we were particularly interested in this variation. We assumed that the short-term changes and fluctuations in income inequality measured both with Gini index and relative poverty were positively associated with the use of antidepressants in the municipalities.

For each case we constructed four models. The first includes the intercept and the municipal Gini coefficient. The second model is calculated for the intercept and the municipal relative poverty rate. The third includes both the Gini coefficient and an indicator of relative poverty, and allowed us to study the effects of these factors vis-à-vis each other. Finally, the fourth model includes all the control variables.

## Results

**Figure 2:**
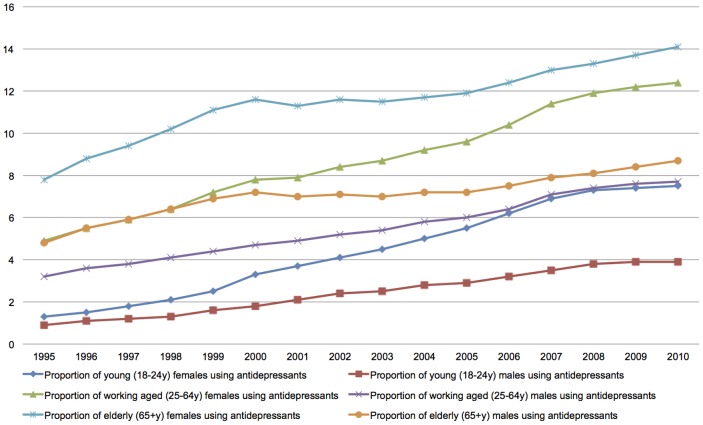
Proportion of antidepressant users by gender and age groups between 1995 and 2010 in Finland.

The proportions of antidepressant users increased in Finland across all age groups and for both males and females between 1995 and 2010 (Figure 2). The most dramatic changes concern young adults. The proportion increased for young males by a multiple of 3.3, and for young females by a multiple of 4.8. The corresponding figures for working aged males and females were 1.4 and 1.5, respectively. Over the 15-year period the proportion for over 65-year-old males and females increased by 81 per cent.

Table 1 gives the descriptive statistics of the variables calculated over the years and municipalities. Tables 2 to 7 present the results from the regression analysis for the three age groups and for both genders.

**Table 2:** GLM analysis of depression among young adult females in Finnish municipalities (1995–2010) with municipality and year as fixed factors.

	Model I		Model II		Model III		Model IV	
	estimate	sign.	estimate	sign.	estimate	sign.	estimate	sign.
Income inequality (Gini)	0.025				0.023		0.016	
Relative poverty			0.057	**	0.055	**	0.083	**
Education level							0.068	**
Not in education or training							0.09	***
Youth unemployment (both male and female)							−0.035	***
R2	79.4		79.4		79.5		80.1	
Observations	2969							

+ p<0.1;

* p< 0.05;

** p< 0.01;

*** < 0.001.

**Table 3:** GLM analysis of depression among young adult males in Finnish municipalities (1995–2010) with municipality and year as fixed factors.

	Model I		Model II		Model III		Model IV	
	estimate	sign.	estimate	sign.	estimate	sign.	estimate	sign.
Income inequality (Gini)	0.015				0.015		0.014	
Relative poverty			0.021		0.02		0.042	**
Education level							0.033	*
Not in education or training							0.032	***
Youth unemployment (both male and female)							0.005	*
R2	71.5		71.5		71.6		71.9	
Observations	2742							

+ p<0.1;

* p<0.05;

^**^ p<0.01;

^***^ <0.001.

**Table 4:** GLM analysis of depression among working-aged females in Finnish municipalities (1995–2010) with municipality and year as fixed factors.

	Model I		Model II		Model III		Model IV	
	estimate	sign.	estimate	sign.	estimate	sign.	estimate	sign.
Income inequality (Gini)	−0.008				−0.008		−0.01	
Relative poverty			−0.01		−0.01		−0.02	
Divorce							0.003	
Education level							−0.009	
Unemployment							0.009	
R2	91.1		91.1		91.1		91.2	
Observations	5152							

+ p<0.1;

* p<0.05;

^**^ p<0.01;

^***^ <0.001.

**Table 5:** GLM analysis of depression among working-aged males in Finnish municipalities (1995–2010) with municipality and year as fixed factors.

	Model I		Model II		Model III		Model IV	
	estimate	sign.	estimate	sign.	estimate	sign.	estimate	sign.
Income inequality (Gini)	−0.008				−0.009		−0.01	
Relative poverty			0.018	**	0.019	**	0.01	
Divorce							−0.003	
Education level							−0.024	**
Unemployment							0.0001	
R2	88.7		88.7		88.7		88.7	
Observations	5153							

+ p<0.1;

* p<0.05;

** p<0.01;

*** <0.001.

**Table 6:** GLM analysis of depression among elderly females in Finnish municipalities (1995–2010) with municipality and year as fixed factors.

	Model I		Model II		Model III		Model IV	
	estimate	sign.	estimate	sign.	estimate	sign.	estimate	sign.
Income inequality (Gini)	−0.045	**			−0.041	**	−0.039	**
Relative poverty			−0.049	**	−0.045	**	−0.083	***
Education level							−0.048	**
Living alone							0.033	**
R2	76.3		76.3		76.3		76.4	
Observations	5139							

+ p<0.1;

* p<0.05;

^**^ p<0.01;

^***^ <0.001.

**Table 7:** GLM analysis of depression among elderly males in Finnish municipalities (1995–2010) with municipality and year as fixed factors.

	Model I		Model II		Model III		Model IV	
	estimate	sign.	estimate	sign.	estimate	sign.	estimate	sign.
Income inequality (Gini)	−0.021	+			−0.02	+	−0.021	+
Relative poverty			−0.008		−0.006	**	−0.005	
Education level							0.01	
Living alone							0.005	
R2	68.1		68.1		68.1		68.2	
Observations	5097							

+ p<0.1;

* p<0.05;

^**^ p<0.01;

^***^ <0.001.

For young adult females, changes in Gini index were not positively associated with changes in the use of antidepressants, while a statistically significant association (p<0.01) was identified concerning changes in relative poverty and use of antidepressants (Table 2). The proportion of young adults using antidepressants increased in municipalities where the number of people living below the relative poverty threshold increased. This association was statistically significant even after control variables were introduced. An increase of one percentage point in relative poverty was found to have increased the use of antidepressants by 0.08 percentage points (Model IV). A weak but statistically significant positive association (p<0.01) between changes in the use of antidepressants and relative poverty was found for young adult males in the adjusted model (IV) (Table 3). A positive association (p<0.001) was discovered for both young adult females and males between changes in the proportion of antidepressant users and the proportion of those not being educated or trained.

Only very few statistically significant associations between changes in the use of antidepressants and other factors were found for working aged individuals (Tables 4 and 5). An Increase in relative poverty was positively associated (p<0.01) with the proportion of working aged males using antidepressants. The association was significant also when Gini index was included, but disappeared once further control variables were added to the model. Changes in the level of education (p<0.01) were negatively associated with changes in the use of antidepressants among working aged males.

The most surprising results were found for the elderly. Statistically significant counter-intuitive associations were discovered for both females and males. The proportion of elderly females using antidepressants decreased with increases in Gini index and relative poverty (Table 6). The association proved robust even after control variables were introduced (p<0.001 in model IV). An increase of one percentage point in relative poverty decreased the use of antidepressants by 0.08 percentage points, while a corresponding increase in Gini coefficient decreased the use of antidepressants by 0.04 percentage points (Model IV). For males, changes in relative poverty were negatively associated with changes in the use of antidepressants (p<0.01 in model III) (Table 7). The association disappeared in the adjusted model. Changes in the proportion of those living alone were positively associated with the use of antidepressants among elderly females (p<0.01), while a negative association was found for changes in level of education and proportion of antidepressant users among elderly females (p<0.01).

To gain more insight into the results for the elderly we ran an additional control model where we included average tax revenue for the municipality, in order to determine if the increase in income inequality could have been caused by an increase in the general income level. Tax revenue was not available in the dataset before 2000. Therefore control models were run from 2000 to 2010. The negative relationship between Gini index and use of antidepressants among elderly females proved robust. (The results are shown in Tables S1 and S2.) However, the negative relationship between relative poverty and the use of antidepressants disappeared for both females and males as changes in the level of tax revenue reduced the effects of the changes in relative poverty. Finally, we carried out another sensitivity test by running the analysis separately for municipalities with more than 10 000 inhabitants (1698 year observations) and for municipalities with more than 1 000 but less than 10 000 inhabitants (3441 year observations)(results not shown here). The negative relationship between inequality and use of antidepressants among elderly females disappeared for the larger municipalities, but not for the medium-sized ones (Gini −0.05 p<0.01, relative poverty −0.12 p<0.001).

## Discussion

Most studies on the health effects of income inequality have been carried out at the population level. Our results have demonstrated large age-group and gender differences in the effects of economic measures and the use of antidepressants. We have shown that the contextual mental health effects of economic changes on young adults may result from the different accumulation of exposures that have their source in the society, namely in the numbers of people living in relative poverty. Given the dramatic changes in the use of antidepressant (Figure 2), raising the prevalence of antidepressants by 0.08 percentage points per one percentage point increase in relative poverty (young females) is a fairly small effect.

Changes in Gini coefficient across municipalities were not positively linked with changes in the use of antidepressants. On the contrary, among elderly females antidepressants were used less in more unequal municipalities. We were also able to show that being outside education and training was positively associated with antidepressant use among young adults, while the proportion of elderly females living alone was positively associated with the use of antidepressants among elderly females.

We used the proportion of antidepressant users as an indicator of depression prevalence. The variable is relatively well suited for use as an indirect indicator of the prevalence of depression [31]. The use of antidepressants has increased in Finland, while the prevalence of depression measured by depression symptom scales has remained almost constant [33]. Further, the use of antidepressants is particularly prevalent among the very old [34]. There was a strong correlation (r = 0,86) between mental health diagnosis for invalidity pension (mostly depression cases) and the use of antidepressant among 18–64 year olds from 2003 to 2012 (www.kela.fi/kelasto).

We may assume that older individuals would be more immune to the negative effects of growing income inequalities within the municipalities, but it is more difficult to explain why inequality would “protect” elderly females from depression. To gain more insight into the questions we inserted lags of one, two and three years into the models. With regard to Gini index and relative poverty there was no consistent pattern of effects for the elderly or for working aged females. For working aged males relative poverty was negatively associated with the use of antidepressants with lags of one, two and three years.

The main question concerns under-use or over-use of antidepressants. Elderly females and working aged males living in poorer municipalities and elderly females living in more unequal municipalities may use fewer medicines due to lack of money and access to doctors [17]. On the other hand, overuse of psychotropics including antidepressants among the elderly has been a topic of concern in Finland [34]. Antidepressants are also prescribed for other indications such as anxiety, chronic pain and sleeping disorders [31]. It is possible that overuse is more frequent in more equal municipalities. A complementary explanation is that non-psychiatric use of antidepressants is less common in more unequal municipalities. It is also possible that the age breakdown of those over 65 years of age has affected the results. To test these additional hypothesis we included a variable on the number of physicians per 10 000 inhabitants (available for years 1995–2007). The variable was used for two age groups: the elderly and the youth. In Finland 90% of employed people are covered through occupational health care, which means that they receive outpatient health care services on those locations where they work. The previous results proved robust showing that poverty and Gini index were positively associated with the use of antidepressants among the youth and negative associated the use of antidepressants among the elderly (not all coefficients were statistically significant). The results indicate that lack of material resources may prevent the elderly from using antidepressants in relatively poor and unequal municipalities in Finland. This rise more concerns with regard to growing inequalities in health [35].

To test non-linear associations we modelled both Gini index and relative poverty with categorical variable (quartile dummies). No considerable non-linear associations of extreme levels of Gini coefficient or relative poverty were discovered.

Clearly, municipal Gini index is not a municipal-level factor that was positively associated with the use of antidepressants in any of the studied age groups in Finland. The direct health effects of within municipality income inequality did not represent generalizable or particular psychological processes that influence the use of antidepressants in Finnish municipalities. In short, our results do not support the stress theory. We found some support for the materialist theory by demonstrating positive relationships between changes in the use of antidepressants among young adults and working aged males. The results thus point to a “weaker” rather than “stronger” version of the psychosocial interpretation of how income inequality affects mental health. The results also indicate that the relationships between inequality and health outcomes may vary greatly across different age groups.

A limitation of our study is that we have no information on the distribution of antidepressant use between psychiatric and non-psychiatric indications among Finnish municipalities. A major limitation of this approach is the possibility of so called ecological fallacy, which resulted from the non-availability of individual level data for this study. Municipalities do not become depressed, individuals do. We were not able to determine if those suffering from economic deprivation were actually the same individuals as those using antidepressants. Multilevel analysis utilizing both individual level longitudinal data on depression measures and local level contextual factors are needed for more detailed analysis.

## Conclusions

Changes in within municipality Gini index were not positively associated with changes in the use of antidepressants in studied Finnish municipalities between 1995 and 2010. More young adult females used antidepressants in municipalities where relative poverty increased, while fewer elderly females used antidepressants in municipalities where Gini index increased.

## Supporting Information

Table S1 GLM analysis of antidepressants' use among elderly females in Finnish municipalities (2000–2010) with year and municipality as fixed factors.
(PDF)

Table S2 GLM analysis of antidepressants' use among elderly males in Finnish municipalities (2000–2010) with year and municipality as fixed factors.
(TIFF)

## References

[1] Kawachi I, Kennedy BP (2002) The Health of Nations: Why Inequality is Harmful for Your Health. New York: The New Press.

[2] Marmot M (2004) Status Syndrome. How your social standing directly affects your health? London: Bloomsbury.

[3] Fritzell J, Lundberg O, editors (2007) Health Inequalities and Welfare Resources. Bristol: Policy Press.

[4] Lahelma E, Lundberg O (2009) Health inequalities in European welfare states. The European Journal of Public Health 19: 445–446. 19770226 10.1093/eurpub/ckp120

[5] Zheng H (2009) Rising U.S. income inequality, gender and individual self-rated health, 1972–2004. Social Science & Medicine 69: 1333–1342. 19733951 10.1016/j.socscimed.2009.08.016

[6] Rowlingson K (2011) Does income inequality cause health and social problems? Joseph Rowntree Foundation. Available: www.jrf.org.uk. Accessed 10 November 2011.

[7] Pop I, Ingen E, Oorschot W (2012) Inequality, Wealth and Health: Is Decreasing Income Inequality the Key to Create Healthier Societies? Social Indicators Research. DOI 10.1007/s11205-012-0125-6. Available: http://link.springer.com/article/10.1007/s11205-012-0125-6#page-1. Accessed 5 March 2014.

[8] Muramatsu N (2003) County-level income inequality and depression among older Americans. Health Services Research 38: 1863–1883. 14727801 10.1111/j.1475-6773.2003.00206.x PMC1360977

[9] Henderson C, Liu X, Diez Roux AV, Link BG, Hasin D (2004) The effects of US state income inequality and alcohol policies on symptoms of depression and alcohol dependence. Social Science & Medicine 58: 565–75. 14652052 10.1016/s0277-9536(03)00228-4

[10] Pickett KE, James OW, Wilkinson RG (2006) Income inequality and the prevalence of mental illness: a preliminary international analysis. Journal of Epidemiology and Community Health 60: 646–7. 16790839 10.1136/jech.2006.046631 PMC2652881

[11] Steptoe A, Tsuda A, Tanaka Y, Wardle J (2007) Depressive symptoms, socio-economic background, sense of control, and cultural factors in university students from 23 countries. International Journal of Behavioral Medicine 14: 97–107. 17926438 10.1007/BF03004175

[12] Wilkinson RG, Pickett KE (2007) The problems of relative deprivation: why some societies do better than others. Social Science & Medicine 65: 1965–78. 17618718 10.1016/j.socscimed.2007.05.041

[13] Friedli L (2009) Mental health, resilience and inequalities: how individuals and communities are affected. Geneva: World Health Organisation.

[14] Messias E, Eaton W, Grooms B (2007) Economic Grand Rounds: Income Inequality and Depression Prevalence Across the United States: An Ecological Study. Psychiatric Services 62: 710–712. 10.1176/ps.62.7.pss6207_0710 21724781

[15] Lorant V, Deliege D, Eaton W, Robert A, Philippot P, et al (2003) Socioeconomic inequalities in depression: a meta-analysis. American Journal of Epidemiology 157: 98–112. 12522017 10.1093/aje/kwf182

[16] Melchiora M, Chastanga J-F, Leclerca A, Ribetc C, Rouillond F (2010) Low socioeconomic position and depression persistence: longitudinal results from the GAZEL cohort study. Psychiatry Research 177: 92–96. 20381167 10.1016/j.psychres.2009.08.002

[17] Butterworth P, Olesen SC, Leach LS (2013) Socioeconomic differences in antidepressant use in the PATH through life study: Evidence of health inequalities, prescribing bias, or an effective social safety net? Journal of Affective Disorders 149: 75–83. 23394713 10.1016/j.jad.2013.01.006

[18] Santiago CD, Wadsworth ME, Stump J (2001) Socioeconomic status, neighbourhood disadvantage, and poverty-related stress: Prospective effects on psychological syndromes among diverse low-income families. Journal of Economic Psychology 32: 218–230.

[19] Wilkinson R, Pickett KE (2009) The Spirit Level: Why More Equal Societies Almost Always Do Better. London: Penguin Books.

[20] Sapolsky RM (1997) The trouble with testosterone: and other essays on the biology of the human predicament. New York: Touchstone.

[21] Lynch J, Davey Smith G, Harper S, Hillemeier M (2004) Is Income Inequality a Determinant of Population Health, Part 2. U.S. National and Regional Trends in Income Inequality and Age- and Cause-Specific Mortality. Milbank Quarterly 82: 355–400. 15225332 10.1111/j.0887-378X.2004.00312.x PMC2690174

[22] Belle D, Doucet J (2003) Poverty, Inequality, and Discrimination as Sources of Depression among U.S. Women. Psychology of Women Quarterly 27: 101–113.

[23] Lundberg O, Yngwe MA, Stjärne MK, Elstad JI, Ferrarini T, et al (2008) The role of welfare state principles and generosity in social policy programmes for public health: an international comparative study. Lancet 372: 1633–40. 18994660 10.1016/S0140-6736(08)61686-4

[24] Mackenbach J, Stirbu I, Roskam A-J, Schaap M, Menvielle G, et al (2008) Socioeconomic Inequalities in Health in 22 European Countries. New England Journal of Medicine 358: 2468–2481. 18525043 10.1056/NEJMsa0707519

[25] Hill AB (1965) The Environment and Disease: Association or Causation? Proceedings of the Royal Society of Medicine 58: 295–300. 14283879 10.1177/003591576505800503 PMC1898525

[26] Galea S, Ahern J, Nandi A, Tracy M, Beard J, et al (2007) Urban Neighborhood Poverty and the Incidence of Depression in a Population-Based Cohort Study. Annals of Epidemiology 17: 3171–179. 10.1016/j.annepidem.2006.07.008 PMC2442459 17320784

[27] Townsend P (2003) Child poverty in the developing world. Bristol: Policy Press.

[28] Kalela J, Kiander J, Kivikuru U, Loikkanen H, Simpura J, editors (2001) Down from the Heavens, Up from the Ashes. The Finnish Economic Crisis of the 1990s in the Light of Economic and Social Research. Helsinki: Government Institute for Economic Research.

[29] OECD (2008) Growing Unequal? Income Distribution and Poverty in OECD. Paris: OECD.

[30] Bambra C (2011) Social inequalities in health: the Nordic welfare state in a comparative perspective. In: Kvist J, Fritzell J, Hvinden B & Kangas O, Changing Social Equality. The Nordic Welfare Model in the 21st Century. Bristol: Policy Press. pp. 143–164.

[31] Sihvo S, Isometsä E, Kiviruusu O, Hämäläinen J, Suvisaari J, et al (2008) Antidepressant utilization patterns and determinants of short-term and non-psychiatric use in the Finnish general adult population. Journal of Affective Disorders 111: 94–105. 18276016 10.1016/j.jad.2008.01.012

[32] Huber E, Stephens JD (2000) Partisan governance, women's employment, and the social democratic service state. American Sociological Review 65: 323–342.

[33] Pirkola SP, Isometsä E, Suvisaari J, Aro H, Joukamaa M, et al (2005) DSM-IV mood-, anxiety- and alcohol use disorders and their comorbidity in the Finnish general population—results from the Health 2000 Study. Social Psychiatry Psychiatric Epidemiology 40: 1–10. 15624068 10.1007/s00127-005-0848-7

[34] Hartikainen S, Klaukka T (2004) Use of psychotropics is high among very old people. European Journal of Clinical Pharmacolpgy 59: 849–850. 10.1007/s00228-003-0702-3 14652704

[35] Shkolnikov V, Andreev E, Jdanov D, Jasilionis D, Kravdal Ø, et al (2012) Increasing absolute mortality disparities by education in Finland, Norway and Sweden, 1971–2000. J Epidemiol Community Health 66: 372–378. 21282141 10.1136/jech.2009.104786

