# Supplementary material for: Is Income Inequality ‘Toxic for Mental Health’? An Ecological Study on Municipal Level Risk Factors for Depression
Source: PLoS One. 2014 Mar 27;9(3):e92775. doi: 10.1371/journal.pone.0092775 (PMC3968015; doi:10.1371/journal.pone.0092775)
Supplement: Table S1 — GLM analysis of antidepressants' use among elderly females in Finnish municipalities (2000–2010) with year and municipality as fixed factors. (PDF) [file pone.0092775.s001.pdf]

Supplemenatary Table 1. GLM analysis of antidepressants' use among elderly females in Finnish municipalities (2000–2010) with year and municipality as fixed factors.

	Model			
	1	2	3	4
	Estimate			
Gini	-0.033*		-0.032*	-0.034*
Poverty		-0.025	-0.022	-0.034
Living alone				0.034*
Tax revenue				-0.0001
R2	76.3	76.3	76.3	76.4
Observations	3548			

+ p<0.1, * p< 0.05, ** p< 0.01, *** < 0.001
