# Supplementary figures and images for: Is Income Inequality ‘Toxic for Mental Health’? An Ecological Study on Municipal Level Risk Factors for Depression
Source: PLoS One. 2014 Mar 27;9(3):e92775. doi: 10.1371/journal.pone.0092775 (PMC3968015; doi:10.1371/journal.pone.0092775)

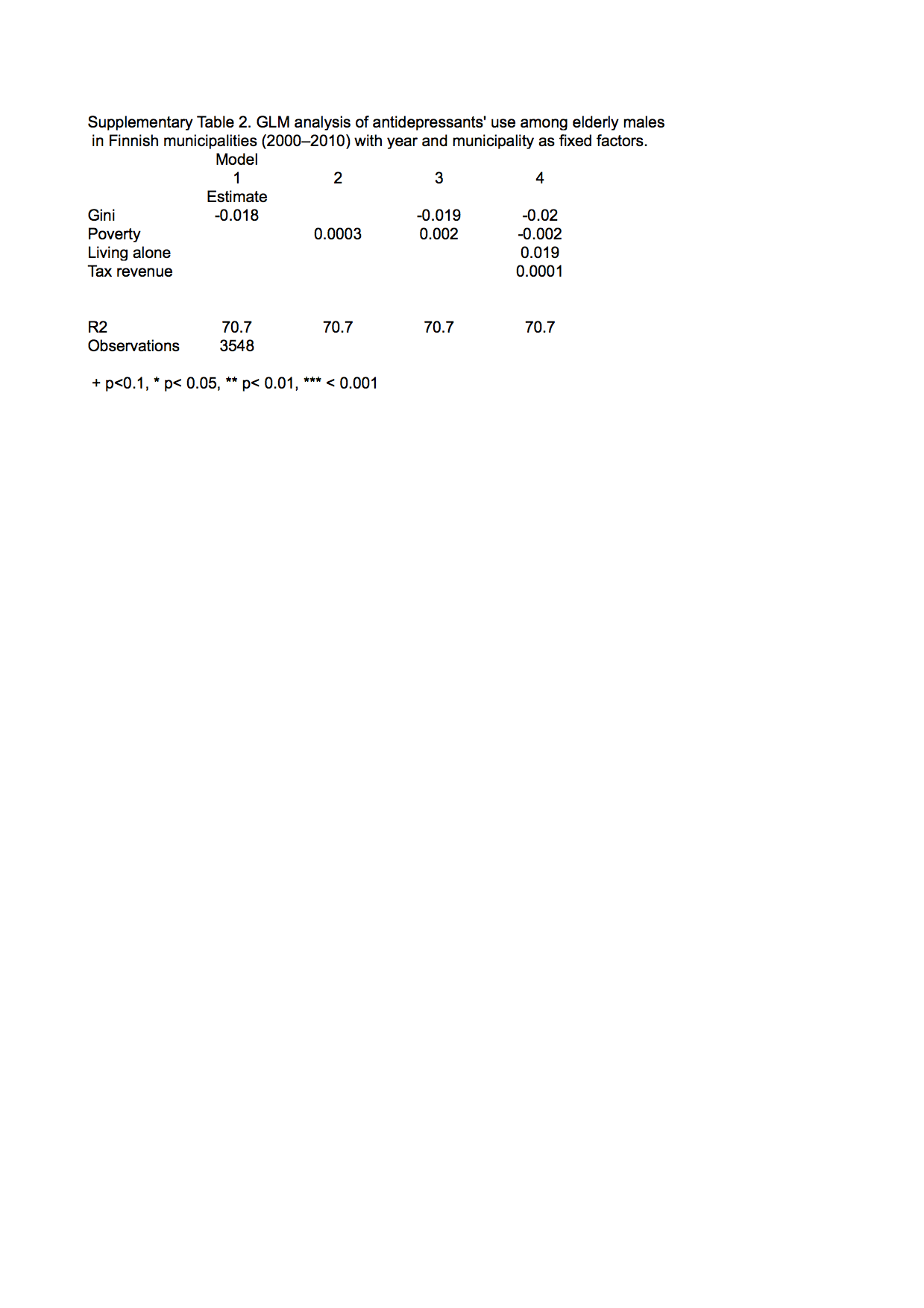

Supplement: Table S2 — GLM analysis of antidepressants' use among elderly males in Finnish municipalities (2000–2010) with year and municipality as fixed factors. (TIFF) [file pone.0092775.s002.tiff]
